# Feasibility and Preliminary Effectiveness of a Peer-Developed and Virtually Delivered Community Mental Health Training Program (Emotional CPR): Pre-Post Study

**DOI:** 10.2196/25867

**Published:** 2021-03-04

**Authors:** Amanda L Myers, Caroline Collins-Pisano, Joelle C Ferron, Karen L Fortuna

**Affiliations:** 1 Department of Public Health Rivier University Nashua, NH United States; 2 Department of Psychiatry Geisel School of Medicine at Dartmouth College Hanover, NH United States; 3 Department of Psychiatry Geisel School of Medicine at Dartmouth College Lebanon, NH United States

**Keywords:** Emotional CPR (eCPR), peer support, peer-delivered training, mental health, community mental health

## Abstract

**Background:**

The COVID-19 pandemic has led to a global mental health crisis, highlighting the need for a focus on community-wide mental health. Emotional CPR (eCPR) is a program and practice developed by persons with a lived experience of recovery from trauma or mental health challenges to train community members from diverse backgrounds to support others through mental health crises. eCPR trainers have found that eCPR may promote feelings of belonging by increasing supportive behaviors toward individuals with mental health problems. Thus, clinical outcomes related to positive and negative affect would improve along with feelings of loneliness.

**Objective:**

This study examined the feasibility and preliminary effectiveness of eCPR.

**Methods:**

We employed a pre-post design with 151 individuals, including peer support specialists, service users, clinicians, family members, and nonprofit leaders, who participated in virtual eCPR trainings between April 20, 2020, and July 31, 2020. Instruments were administered before and after training and included the Herth Hope Scale; Empowerment Scale; Flourishing Scale (perceived capacity to support individuals); Mindful Attention Awareness Scale; Active-Empathic Listening Scale (supportive behaviors toward individuals with mental health challenges); Social Connectedness Scale (feelings of belonging and connection with others); Positive and Negative Affect Schedule; and University of California, Los Angeles 3-item Loneliness Scale (symptoms and emotions). The eCPR fidelity scale was used to determine the feasibility of delivering eCPR with fidelity. We conducted 2-tailed paired *t* tests to examine posttraining improvements related to each scale. Additionally, data were stratified to identify pre-post differences by role.

**Results:**

Findings indicate that it is feasible for people with a lived experience of a mental health condition to develop a program and train people to deliver eCPR with fidelity. Statistically significant pre-post changes were found related to one’s ability to identify emotions, support others in distress, communicate nonverbally, share emotions, and take care of oneself, as well as to one’s feelings of social connectedness, self-perceived flourishing, and positive affect (*P*≤.05). Findings indicated promising evidence of pre-post improvements (not statistically significant) related to loneliness, empowerment, active-empathetic listening, mindfulness awareness, and hope. Nonprofit leaders and workers demonstrated the greatest improvements related to loneliness, social connectedness, empathic listening, and flourishing. Peer support specialists demonstrated the greatest improvements related to positive affect, and clinicians demonstrated the greatest improvements related to mindfulness awareness.

**Conclusions:**

Promising evidence indicates that eCPR, a peer-developed and peer-delivered program, may increase feelings of belonging while increasing supportive behaviors toward individuals with mental health problems and improving clinical outcomes related to positive and negative affect and feelings of loneliness.

## Introduction

To date, the United States has had over 22.8 million confirmed COVID-19 cases and over 500,000 associated deaths [1]. During the COVID-19 pandemic, rates of individuals experiencing psychological distress increased, which has happened during prior outbreaks of infectious diseases [2]. Over 1 in 3 adults in the United States reported symptoms of anxiety or depressive disorders since the onset of the COVID-19 pandemic [3]. Further, social isolation, loneliness, and fear, as well as significant life events during the pandemic (eg, the loss of a job or loved one), are all risk factors for suicidality [4]. The United Nations has called for widespread mental health psychoeducation to identify, understand, and support people outside of clinical environments [5].

Community mental health psychoeducation training programs have been disseminated internationally and have been shown to increase participants’ knowledge regarding mental health, decrease negative attitudes about mental health care, and increase supportive behaviors toward individuals with mental health problems among a diverse group of trainees (eg, adults, youth and teens, public safety officials, first responders, veterans, rural community members, students in higher education, and older adults) [6]. Service users, advocates, provider organizations, policy makers, and researchers concur that widespread psychoeducation is essential for communities to identify, understand, and respond to signs of mental health conditions.

Substantial progress has been made in widespread psychoeducation among community members by making people aware of different mental health conditions, and it has shown effectiveness related to increased knowledge, social attitudes, and helping behaviors toward individuals with mental health challenges [7]. However, it is not known if it is effective in assisting a person through an emotional crisis in the moment. Emotional CPR (eCPR) has the potential to provide people with the skills needed to assist persons through emotional crises.

Community mental health psychoeducation trainings may offer a therapeutic component to facilitate the development of supportive behaviors toward individuals with mental health challenges in addition to clinical outcomes. People who have a lived experience of a mental health condition could be an important asset in these situations by sharing their lived experiences and recovery journeys and assisting in the development of trainings, yet the impact of such an approach is not known. eCPR is designed to educate individuals on mental health challenges and teaches the public how to support others through feelings of mental health distress, address stigmatizing attitudes toward oneself and others, and offer social support.

eCPR is a community mental health psychoeducation virtual training program that was developed to increase supportive practices in community settings and developed to be used by both clinical and nonclinical community members. Specifically, eCPR, a peer-developed and peer-delivered manualized program, aims to teach the public how to (1) address mental health challenges, (2) support others as they work through feelings of mental health distress, (3) address stigmatizing attitudes toward oneself and others, and (4) offer social support. The purpose of this study was to examine the feasibility and preliminary effectiveness of eCPR by exploring pre-post changes in self-perceived levels of hope, empowerment, flourishing, mindful awareness, empathic listening skills, social connectedness, positive and negative affect, and feelings of loneliness among eCPR trainees. This is the first study conducted to examine both the feasibility and preliminary effectiveness of the eCPR training program on participants and the feasibility and effectiveness of delivering the eCPR training over a virtual platform.

## Methods

### Peer-Academic Partnership

This study employed the Peer-Academic Partnership [8] to explore the preliminary effectiveness of eCPR. The Peer-Academic Partnership is a community-engaged research approach based on 9 principles of community engagement set by the Centers for Disease Control [9]: (1) develop a clear understanding of the purpose, goal, and population involved in community change; (2) become knowledgeable about all aspects of the community; (3) interact and establish relationships with the community; (4) encourage community self-determination; (5) partner with the community; (6) respect community diversity and culture; (7) activate community assets and develop capacity; (8) maintain flexibility; and (9) commit to long-term collaboration. The partnership engages peer support specialists (ie, people with mental health challenges, trained and accredited by their respective state to offer Medicaid-reimbursable support services), people with lived experiences of mental health challenges, and peer and nonpeer scientific researchers in the development and implementation of research studies important to the mental health community.

### Description of eCPR

eCPR is a training developed by persons with a lived experience of a mental health condition and delivered by peer support specialists using a manualized workbook. This training has been offered internationally since its development over 10 years ago, yet this is the first empirical study of eCPR. The term “eCPR” is based on the original use of the term “CPR.” Where traditional CPR revitalizes a person’s physical heart, eCPR is intended to revitalize one’s emotional heart. eCPR focuses on connecting, empowering, and revitalizing individuals in community settings. eCPR is based on the recovery model of mental health and guided by principles of recovery. Principles of recovery include hope, person-driven treatment, relationships, culture, multiple pathways, addressing of trauma, a holistic approach, development of strengths and responsibilities, peer support, and respect [10]. Scientifically, recovery has been found to be associated with increased empowerment, hope, and quality of life and decreased incidence of psychiatric symptoms and loneliness [11].

eCPR was developed using an iterative design process based on the experiences of an expert panel of peer support specialists, nonprofit leaders, and people with mental health challenges through the National Empowerment Center, a peer-run nonprofit organization. Although eCPR has been offered for the past 10 years in person by a worldwide network of trainers, the COVID-19 pandemic prompted conversion to an online delivery of the training. Since April 2020, eCPR trainings have been delivered virtually using Health Insurance Portability and Accountability Act (HIPAA)–compliant videoconference software over 3 days of 4 hours of training (12 hours total) by 2 trainers who identify as individuals with a lived experience of a mental health condition. Trainers completed 60 hours of training on eCPR, which included education and simulation-based training and a performance test. Between sessions, trainers assign eCPR trainees homework, which includes assigned readings from the online or mailed versions of the eCPR manual and practice exercises of newly learned skills with friends and among themselves. eCPR includes the following evidence-based principles to support recovery in the community: peer support, coping skills training, and psychoeducation. A total of 7 training modules are completed through group experiential learning, didactic learning, role-play, and dialogical formats (Textbox 1).

**Training modules in Emotional CPR (eCPR).**

**Training modules**

1. Connecting with others:** **Participants are taught how to connect through feelings first, engaging all their senses and opening their heart while respecting each other as equally human. They learn the value of engaging in emotional dialogue through expressing and responding to each other’s feelings.

2. Using nonverbal communication:** **Participants learn to connect without asking questions or pursuing a story through emphasis on nonverbal dimensions of communication, such as facial expression, gestures, tone of voice, and description of bodily sensations. 

3. Cultural empathy across worldviews: Participants learn to validate another person’s worldview by entering their frame of reference and sensing the inner meaning. This includes not only awareness of cultural differences but interconnected social categorizations, such as race, class, gender, and whatever way a person defines themselves. 

4. Learning a trauma-informed approach: Participants learn about possible causes and types of trauma as well as ways to heal trauma, understanding that trauma causes alienation, disempowerment, and emotional numbing, while eCPR creates emotional connection, empowerment, and revitalization. 

5. Addressing feelings of mental distress and thoughts of suicide: Participants discuss crisis situations, including suicidal thoughts and behaviors, acute stress reactions, and extreme or altered states of consciousness, as well as ways to help.

6. Empowerment: Participants learn that they can facilitate empowerment by being with a person in distress in such a manner that they respect that the person has a healer within and refrain from judging, fixing, or planning for the person.

7. Revitalization:** **Participants learn that mutual revitalization occurs through deep emotional connection, which is experienced by all participants as increased energy, life, creativity, and hope. 

Group trainings include individuals from a variety of backgrounds and experiences to promote diversity of perspectives and experiential learning through interacting with one another outside of a clinical environment. For example, one eCPR training could potentially include caregivers, emergency workers (eg, firefighters, hospital staff), peer support specialists, family of individuals with mental health challenges, and clinicians.

A total of 56 eCPR trainings occurred virtually via a HIPAA-compliant videoconferencing platform between April 20, 2020, and July 31, 2020, and included an average of 10 participants. Using a pre-post, single-arm design, individuals were asked to complete online surveys to examine the impact of the eCPR training on themselves. No incentive was provided. The Dartmouth College Institutional Review Board approved this study.

### Instruments

Online surveys were administered both prior to the eCPR training and upon its conclusion. Trainers sent the pretraining survey link via email both 1 week prior to the start of the training and the day before the training. At the conclusion of each training, trainers sent the posttraining survey link to participants via email. Reminders to complete the surveys were emailed to participants in the days following each training. Items included in the surveys were selected to reflect key goals described in the research literature on peer support, including engendering hope, facilitating empowerment, and providing social support. The surveys also included eCPR-specific items designed using the Peer-Academic Partnership through a series of meetings with the principal investigator (KLF). These items were designed based on what the National Empowerment Center views as the most crucial elements and aims of eCPR (including trauma-informed support, the act of being with another person in crisis, communication, and self-care) and aimed to explore participants’ agreement with statements related to emotional support. Sample questions include “I feel comfortable being with another person and listening to them,” “I can sit with another person and let them express strong emotions,” “Trauma plays a significant role in people's emotional states,” “I recognize nonverbal ways others communicate,” and “I know how to take care of myself before and after being with someone in distress.” Response options include “strongly agree,” “agree,” “disagree,” “neither agree or disagree,” and “strongly disagree.”

### Perceived Capacity to Support Individuals

To measure perceived capacity to support individuals, we incorporated 3 scales. To measure empowerment, we used the Empowerment Scale [12,13], which was developed with service users with serious mental illness and is a widely used, valid, reliable 28-item scale that measures empowerment [13]. Sample questions include “I can pretty much determine what will happen in my life” and “People are only limited by what they think is possible.” Response options include “strongly agree,” “agree,” “disagree,” and “strongly disagree.” Scores were totaled and averaged, with a potential score of 1 through 4, in which lower scores indicated higher levels of empowerment.

The Herth Hope scale was used to measure (1) inner sense of temporality and future, (2) inner positive readiness and expectancy, and (3) interconnectedness with self and others [14]. This scale was established as reliable and valid for both elderly and ill populations [14]. Sample questions include “I have a positive outlook toward life,” “I have short- and/or long-range goals,” and “I feel scared about my future.” Response options include “strongly agree,” “agree,” “disagree,” and “strongly disagree.” Two items, “I feel scared about my future” and “I feel all alone,” were reverse scored. Scores were totaled and averaged, with a potential score of 1 through 4, in which lower scores indicated higher levels of hope.

The Flourishing Scale [15] was used to measure self-perceived success in important areas such as (1) relationships, (2) self-esteem, (3) purpose, and (4) optimism. The scale has good psychometric properties and is strongly associated with other psychological well-being scales [16]. The Flourishing Scale has been deemed both reliable and valid for measuring psychosocial functioning in adults, including those with mental and physical health challenges [16]. Sample questions include “I lead a purposeful and meaningful life,” “I am engaged and interested in my daily activities,” and “I am competent and capable in the activities that are important to me.” Response options include “strongly disagree,” “disagree,” “slightly disagree,” “neither agree nor disagree,” “slightly agree,” “agree,” and “strongly agree.” Scores were totaled, with a potential score of 8 through 56, in which a high score indicated a person with many psychological resources and strengths.

### Supportive Behaviors Toward Individuals With Mental Health Problems

To measure supportive behaviors toward individuals with mental health problems, we incorporated 2 scales. The Mindful Attention Awareness Scale is a 15-item scale designed to measure core characteristics of dispositional mindfulness [17]. The scale was established as a valid measure of receptive attention to and awareness of present experience [17]. Sample questions include “I could be experiencing some emotion and not be conscious of it until some time later” and “I tend not to notice feelings of physical tension or discomfort until they really grab my attention.” Response options include “almost always,” “very frequently,” “somewhat frequently,” “somewhat infrequently,” “very infrequently,” and “almost never.” Scores were totaled and averaged, with a potential score of 1 through 6, in which higher scores reflected higher levels of dispositional mindfulness.

The Active-Empathic Listening Scale measures active-empathic listening. Active-empathic listening is the active and emotional involvement of a listener during a given interaction [18]. The multidimensional scale was established as a valid measure of interpersonal communication [18]. Sample questions include “I am sensitive to what others are not saying,” “I am aware of what others imply but do not say,” “I listen for more than just the spoken words,” “I understand how others feel,” and “I show others that I am listening by my body language.” Response options include “always or almost always true,” “usually true,” “often true,” “occasionally true,” “sometimes true,” “usually not true,” and “never or almost never true.” Scores were totaled and averaged, with a potential score of 1 through 7, in which lower scores indicated higher levels of active-empathic listening.

### Feelings of Belonging and Connection With Others

To measure feelings of belonging and connection with others, we used the revised Social Connectedness Scale [19]. This 20-item scale was derived from the shorter Social Connectedness Scale. This scale was deemed valid, though it has been said to have psychometric limitations and possibilities of response bias [19]. Sample questions include “I feel distant from people,” “I am able to relate to my peers,” and “I find myself actively involved in people’s lives.” Response options include “strongly disagree,” “disagree,” “mildly disagree,” “mildly agree,” “agree,” and “strongly agree.” The negatively worded items were reverse scored and summed together with the positively worded items, with a potential score of 20 to 120. Higher scores indicated a stronger sense of social connectedness.

### Symptoms and Emotions

To measure symptoms and emotions, we incorporated 2 scales. The Positive and Negative Affect Schedule (PANAS) [20] is a mood scale that was used to measure positive and negative affect. PANAS was established as a reliable and valid measure of the positive and negative affects of mood. The scale lists mood descriptors, including “interested,” “distressed,” “ashamed,” and “active.” Participants were asked to “indicate the extent you have felt this way over the past week,” with response options including “very slightly or not at all,” “a little,” “moderately,” “quite a bit,” and “very much.” The negatively worded items were reverse scored and summed together with the positively worded items, with a potential score of 20 to 100. Higher scores indicated higher levels of positive affect and lower scores indicated higher levels of negative affect.

To measure loneliness, we used the University of California, Los Angeles (UCLA) 3-item Loneliness Scale [21]. This widely used and valid 3-item scale was derived from the longer 20-item UCLA Loneliness Scale that was deemed not suitable for telephone interviews [21]. Questions include “How often do you feel that you lack companionship?” “How often do you feel left out?” and “How often do you feel isolated from others?” Response options include “hardly ever,” “some of the time,” and “often.” Scores were totaled and averaged, with a potential score of 1 through 3, in which lower scores indicated lower levels of loneliness.

### Fidelity Assessment

The principal investigator monitored intervention fidelity through (1) biweekly discussions between the principal investigator and lead trainers’ supervisors, (2) the trainer’s self-report fidelity tool, and (3) confirmation that participants received an accompanying eCPR training manual (either online or through the mail).

### Procedures

#### Recruitment

At a time that in-person peer support organizations were seeking new ways to bridge the new and sudden social distancing guidelines, trainers reached out to individuals and organizations they had been working with for years to let them know about this opportunity. In addition to reaching out to networks, an invitation was posted on the eCPR website for people to express their interest in participating in the eCPR trainings.

#### Informed Consent

Prior to engaging in the presurvey, participants were given a consent statement on Qualtrics (Qualtrics International). Interested individuals were given the opportunity to meet with the principal investigator to ask questions and review the informed consent form and to contact the principal investigator at any time to withdraw their participation.

### Statistical Analyses

Descriptive statistics were conducted to describe demographic characteristics of the study sample. We conducted 2-tailed paired sample *t* tests to assess the difference between prescores and postscores for statistical significance. All incomplete survey responses were excluded from analyses. Descriptive statistics and analyses were computed using IBM SPSS software (IBM Corp) [22].

## Results

The study included 151 adults aged 18 years and older who identified as peers and service users with a lived experience of any mental health condition, as well as hospital staff, family members, clinicians, nonprofit leaders, and nonprofit workers (Table 1). Inclusion criteria were all members of the community who (1) were 18 years or older; (2) self-reported experiencing any mental health condition or were hospital staff, family members of individuals with mental or physical health conditions, clinicians, nonprofit leaders, nonprofit workers, and all other members of the community; and (3) were able to provide informed consent. Exclusion criteria were as follows: (1) individuals younger than 18 years; (2) individuals deemed cognitively impaired and who were unable to provide consent, identified as not being able to log in to the training; and (3) potential participants who were cognitively impaired and had designated legal guardians.

In total, 560 individuals participated in any portion of the 56 virtual eCPR trainings offered between April 20, 2020, and July 31, 2020. An online website posting reached 64 of the 560 (11.4%) individuals who participated in the trainings. The remainder of the participants were invited through their organizations or through direct emails.

Of the participants who attended the training, we obtained pre-and postsurveys from individuals who completed the full 12 hours of training and completed both the presurvey and postsurvey. Out of the 560 training participants, we received a total of 452 presurvey responses and 318 postsurvey responses. Individuals who did not complete both the pre- and postsurveys in full were excluded from analyses. Of the 560 training participants, the total sample of individuals who completed both a presurvey and postsurvey was 151 participants. Of the final sample of participants (N=151), 81 (53.6%) identified as peer support specialists, 44 (29.1%) were individuals with a lived experience of a mental health challenge, 10 (6.6%) were family members of individuals with mental health challenges, 8 (5.3%) were nonprofit leaders or worked for a nonprofit, and 8 (5.3%) were clinicians. No differences were noted in participants who completed both a presurvey and postsurvey compared with individuals who only completed 1 survey (presurvey or postsurvey). The majority of participants were women (116/151, 77.3%). The majority of the participants were within the age range of 27 to 49 years (71/151, 47.1%), followed by 50 to 64 years (55/151, 36.4%). Of the 151 participants, 115 (77.7%) identified as White, 13 (8.8%) as Black, 4 (2.7%) as American Indian or Alaska Native, 4 (2.7%) as Asian, and 12 (8.1%) as more than one race (Table 2).

Before attending the eCPR training, 151 participants reported their eCPR abilities on a scale of 1 to 5 (1 being the highest [“strongly agree”] and 5 being the lowest [“strongly disagree”]). The average pretraining score was 2.17. After participating in the training sessions, the same group of participants reported an increase in their eCPR abilities and reported an average posttraining score of 2.02 (*P*<.001).

**Table 1:** eCPR participant demographics.

Characteristics		Participants, n (%) (N=151)
**Gender**		
	Male	32 (21.3)
	Female	116 (77.3)
	Other	2 (1.3)
**Age (years)**		
	19-26	16 (9.9)
	27-49	71 (47.1)
	50-64	55 (36.4)
	65+	10 (6.6)
**Race**		
	White	115 (77.7)
	Black/African American	13 (8.8)
	American Indian/Alaska Native	4 (2.7)
	Asian	4 (2.7)
	Native Hawaiian/Pacific Islander	0 (0.0)
	More than one race	12 (8.1)
**Ethnicity**		
	Hispanic/Latino	14 (9.5)
	Non-Hispanic/Latino	134 (90.5)
**Employment status**		
	Full-time	77 (51.3)
	Part-time	42 (34.2)
	Volunteer	11 (7.9)
	Unemployed	15 (3.9)
	Retired	6 (4.0)
**Role**		
	Service user	44 (29.1)
	Peer support specialist	81 (53.6)
	Family member	10 (6.6)
	Clinician	8 (5.3)
	Nonprofit leader or worker	8 (5.3)

**Table 2:** eCPR pre- and postsurvey responses by item.

eCPR^a^ item	Presurvey, mean (SD) (N=151)	Postsurvey, mean (SD) (N=151)	*P* value	Cohen *d*^b^
1. When I see someone in emotional distress, I usually have a good sense about what's wrong, what they need, and how to help them.	2.72 (0.897)	2.58 (1.029)	.11	0.13
2. Support is helping a person solve their problems.	3.41 (1.043)	3.81 (1.137)	<.001*	–0.34
3. Support is about trying to be with another person.	2.13 (0.946)	1.47 (0.757)	<.001*	0.62
4. Trauma plays a significant role in people's emotional states.	1.36 (0.582)	1.28 (0.582)	.17	0.11
5. When I see someone in emotional distress, I want to help them, but don't know how.	3.05 (0.925)	3.74 (0.833)	<.001*	–0.60
6. I can identify my emotions.	1.93 (0.713)	1.73 (0.688)	.002*	0.26
7. I can sit with another person and let them express strong emotions.	1.58 (0.719)	1.39 (0.541)	.003*	0.24
8. I am willing to share my emotions with another person who may be in distress.	1.95 (0.703)	1.60 (0.624)	<.001*	0.42
9. When I'm with someone in distress, I try to figure out solutions for them.	3.15 (1.022)	3.69 (0.967)	<.001*	–0.44
10. I know what it means to “be with” another person.	2.08 (0.728)	1.36 (0.509)	<.001*	0.91
11. I recognize nonverbal ways I have of communicating.	1.91 (0.683)	1.54 (0.651)	<.001*	0.49
12. I feel comfortable being with another person and listening to them.	1.59 (0.667)	1.36 (0.495)	<.001*	0.38
13. I know how to take care of myself before and after being with someone in distress.	2.31 (0.926)	1.80 (0.760)	<.001*	0.61
14. I am open to new ideas and ways of doing them.	1.44 (0.511)	1.34 (0.555)	.06	0.16
15. I recognize nonverbal ways others communicate.	1.92 (0.661)	1.54 (0.551)	<.001*	0.53

^a^eCPR: Emotional CPR.

^b^Cohen *d* measures effect size (0.20=small effect, 0.5=medium effect, 0.8=large effect).

*Statistically significant (*P*≥.05).

Before attending this training, 151 participants reported their perceived capacities to support individuals by responding to the Herth Hope Scale, Empowerment Scale, and Flourishing Scale. There was no significant difference in levels of hope reported before and after the training sessions. However, we did observe an improvement between pretraining and posttraining scores. The average pretraining score was 20.38. The same group of participants reported an average posttraining score of 19.97, resulting in an improvement of 0.41 (*P*=.11). Similarly, there was no significant difference between the average levels of empowerment reported before and after the training sessions. The average pretraining score was 2.15, and after participating in the eCPR training, the same participants reported an average posttraining score of 2.13, resulting in an improvement of 0.02 (*P*=.10). Statistically significant differences were observed in questions related to self-perceived flourishing. Participants demonstrated an average pretraining score related to flourishing of 16.53. After participating in the training sessions, the same group of participants reported an increase in their self-perceived success and reported an average posttraining score of 15.58 (*P*=.008) (Table 3).

Regarding supportive behaviors toward individuals with mental health challenges, 151 participants reported their mindfulness abilities and active-empathic listening skills. There were no statistically significant differences in mindfulness abilities or active-empathic listening skills observed between the pre- and posttraining surveys, but posttraining improvements were observed related to both mindfulness and listening skills. Participants demonstrated an average mindfulness awareness pretraining score of 3.99. After the eCPR training sessions, the same group of participants reported an average posttraining score of 3.87, resulting in an improvement of 0.12 (*P*=.09). In regard to active-empathic listening, participants reported an average pretraining score of 2.41. After the eCPR training sessions, participants reported an average improvement of 0.09 and an average posttraining score of 2.32 (*P*=.11).

Regarding feelings of belonging and connection with others, 151 participants reported their feelings of social connectedness. The average pretraining score was 51.28. After participating in the training sessions, the same group of participants reported an increase in social connectedness and reported an average posttraining score of 48.59 (*P*=.002).

**Table 3:** eCPR training pre-post changes by measure.

Measure	Presurvey, mean (SD) (N=151)	Postsurvey, mean (SD) (N=151)	*P* value	Cohen *d*^a^
Herth Hope Scale	20.38 (2.96)	19.97 (3.29)	.11	0.13
Empowerment Scale	2.15 (0.19)	2.13 (0.21)	.10	0.13
Flourishing Scale	16.53 (5.92)	15.58 (6.08)	.008*	0.22
Mindful Attention Awareness Scale	3.99 (1.03)	3.87 (1.13)	.09	0.14
Active-Emphatic Listening Scale	2.41 (0.77)	2.32 (0.78)	.11	0.13
Social Connectedness Scale	51.28 (14.77)	48.59 (14.69)	.002*	0.25
PANAS^b^	72.18 (11.94)	76.42 (12.32)	<.001*	–0.48
UCLA^c^ Loneliness Scale	1.75 (0.57)	1.69 (0.57)	.12	0.13

^a^Cohen *d* measures effect size (0.20=small effect, 0.5=medium effect, 0.8=large effect).

^b^PANAS: Positive and Negative Affect Schedule.

^c^UCLA: University of California, Los Angeles.

*Statistically significant (*P*≥.05).

Regarding symptoms and emotions, participants (N=151) reported positive and negative affect and levels of loneliness. The average pretraining score related to affect was 72.18. After participating in the training sessions, the same group of participants reported an increase in their positive affect and reported an average posttraining score of 76.42 (*P*<.001). Although there was no significant difference between the average level of loneliness reported before and after the training session, we observed an improvement in scores. The average pretraining score was 1.75. After participating in the eCPR training sessions, the same group of participants reported a decrease in their loneliness and reported an average posttraining score of 1.69 (*P*=.12).

When compared to the pre-post changes observed in other roles, nonprofit leaders and workers reported the greatest improvements in self-perceived flourishing (2.12-point improvement), active-empathic listening abilities (0.5-point improvement), social connectedness (6.5-point improvement), and loneliness (0.37-point improvement). Clinicians reported the greatest improvements in self-reported mindfulness abilities (0.56-point improvement). Additionally, peer support specialists reported the greatest improvements in positive affect (5.10-point improvement).

## Discussion

### Summary of Findings

To the authors’ knowledge, this is the first study to date that aimed to explore the feasibility and preliminary effectiveness of online eCPR. Our findings suggest that eCPR is feasible, as it can be delivered through an online platform with fidelity by peer support specialists. Promising evidence indicates that eCPR, a peer-delivered program, may promote social connectedness by increasing supportive behaviors toward individuals with mental health challenges and improving potential clinical outcomes related to positive and negative affect and feelings of loneliness. Of note, the impact of eCPR may be greater in person and reach a different audience than online eCPR. Exploring these differences in future studies after the COVID-19 lockdown measures and social distancing is an important next step.

The feasibility of eCPR delivery was determined by peer support specialists’ high fidelity scores. Of note, the eCPR training and related manual may have supported peer support specialists’ proficiency in integrating standardized evidence-based intervention components. This study highlights promising findings that a peer-developed and peer-delivered program may support fidelity-adherent mental health psychoeducation and potentially support individuals outside of clinical environments or between clinical encounters. As eCPR training was offered virtually, it is possible this delivery method may reach audiences that would not have otherwise been able to attend an in-person training (ie, individuals with physical limitations, transportation issues, etc).

eCPR may increase feelings of belonging and connection with others. eCPR offers another perspective than the traditional medical model of treatment, as it has a human-centered and trauma-informed approach that focuses on individuals sharing lived experiences with people from varying backgrounds. The practice of eCPR focuses on encouraging individuals to express their feelings and share their lived experiences and perspectives to learn from one another, which may impact perceived and real stigma. Exploring the impact of eCPR on stigma may be an important future study.

eCPR may increase supportive behaviors toward individuals with mental health problems, particularly among clinicians in terms of mindfulness awareness. eCPR demonstrates the value of being with one another, or mindful awareness, and actively listening to the individual who is experiencing mental health problems or a mental health crisis. This trauma-informed approach creates a safe environment for people to experience mental health challenges without fear of involuntary hospital commitment. This type of support is not focused on being solution-driven, which is a common goal of clinicians; rather, eCPR focuses on creating a supportive environment to experience and share feelings and emotions. In this approach, eCPR trainees are coached to trust that the solutions lie within the person being helped.

eCPR is related to the development of new skills to support individuals, and it impacts a person’s emotions. Interestingly, eCPR demonstrated potential effectiveness related to positive and negative affect and a reduction of feelings of loneliness across groups. eCPR’s approach promotes the integration of diverse groups in regard to race, age, roles, and lived experiences. Diversity among group members provides an opportunity to learn from one another’s experiences and perspectives. eCPR’s ability to help individuals provide support to one another is not dependent on whether the individuals are trained mental health clinicians; rather, people from varying educational backgrounds can provide services to one another in a group environment, which may help them relate or grasp new situations related to work with others with mental health challenges.

### Limitations

This study is not without limitations and the results should be interpreted with caution. First, the study design limits the findings and does not allow us to determine the causality of eCPR on outcomes. However, this design was consistent with the purpose of the study, which was to explore feasibility and preliminary effectiveness. As feasibility and preliminary effectiveness have been established, future studies can consider increasing the methodological quality of the study designs. Second, all the trainings studied were online; thus, generalizability is limited to online environments. A scientifically rigorous study exploring barriers and facilitators to eCPR implementation in digital environments may increase understanding of the ease of its implementation. The sample size is too small for subgroup analysis (ie, analysis by role, diagnosis, age, race, etc) but is large enough for the purposes of this study. We do not have further data on why the remaining surveys were incomplete or not completed at all, since the surveys were completely voluntary and participation in the surveys for research purposes did not impact one’s ability to participate in the trainings. Additionally, we have not tracked data on who completed the full 12 hours.

It is unknown whether the improvement in eCPR training participants’ scores is indicative of a change in real-life practice and behavior. Future studies should examine the impact of the training on whether participants exhibit these changes in social connectedness, eCPR skills, mindfulness awareness, and active-empathic listening in everyday practice with others in their community, clinical practice, and social circles. Additionally, further research can be done to examine the impacts of increased hope, positive affect, flourishing, and decreased loneliness on the daily life of eCPR training participants.

Unfortunately, we were unable to determine clinical significance, as comparing effect sizes with equipoise between different studies is not possible due to the heterogeneity of the sample in the current study (ie, nonprofit leaders and workers, clinicians, and peer support specialists). Adequately powered future eCPR studies should explore the clinical significance of the findings to determine real-world outcomes.

### Conclusions

To the authors’ knowledge, this is the first study to examine the feasibility and preliminary effectiveness of a peer-developed and peer-delivered community mental health psychoeducation training. Promising evidence indicates that eCPR, a peer-delivered training, may increase feelings of belonging and closeness with the social world while increasing supportive behaviors toward individuals with mental health problems and improving clinical outcomes related to positive and negative affect and feelings of loneliness. eCPR has shown promising evidence that support services are not limited to a clinician’s office; rather, we can help to heal our community using the skills that are taught throughout the sessions.

## Abbreviations

eCPR: Emotional CPR
HIPAA: Health Insurance Portability and Accountability Act
PANAS: Positive and Negative Affect Schedule
UCLA: University of California, Los Angeles
